# Who Did It?

**Published:** 1887-01-08

**Authors:** Laura Bainbridge


### Who Did It?

By Miss LAURA BAINBRIDGE.

" Poison"——

After the most cursory examination of the patient, young Mr. Tottenham raised his head and distinctly pronounced the word, while he fixed his keen eyes on the faces of the three women standing by the bedside, that he might judge the effect his announcement had on each ; and whether he could detect signs of guilty confusion on any one of the countenances.

To his astonishment, instead of noting such signs on one of the faces, it seemed to him they were visible on all, and in the nervous workings of their hands. Mrs. Spencer, the wife of the senseless, though still living, old man in the bed, gave a gasp as though about to speak, and then hastily caught her underlip between her teeth, and twisted the bracelets on her slender wrists backwards and forwards.

Mrs. Jennings, the respectable middle-aged serving woman next her, clasped and unclasped her hands to pass one across her lips, as though to hide their tremulous twitchings.

Araminta Mabel Tomkins, the kitchen-maid, stood with round eyes staring, and wide open mouth, while her rosy face passed through every stage of pallor, till it arrived at being green.

Mr. Tottenham in his own mind confessed himself fairly baffled; and the while he busied himself with every effort to restore the unconscious man, he was deeply pondering the probabilities of the case. Nothing would have been easier than to draw the conclusion that Mr. Spencer, whom he knew to be a severe sufferer at times, had either intentionally, or otherwise, taken an overdose of some narcotic, which had gone near to emancipating him from the burden of the flesh. Granting this hypothesis, what was the meaning of the conscious looks he had observed? He prided himself on his acumen and power of reading human countenances, and he was ready to stake his professional reputation on the fact that each one of those women had some knowledge of the circumstances ; and he should make it his business, later on, to find out what that knowledge might be. It was scarcely credible that Mrs. Spencer, who appeared the gentlest of women, should have committed a horrible crime, and taken her two servants into her confidence. He had only been a month in the village, as assistant to Dr. Marsh, and had never been to the Spencers' house before : but, from hearsay he had already gathered that Mrs. Spencer bore the character of the most devoted wife ; only on rare occasions leaving the side of the suffering, ill-tempered old man she had married.

Next time he looked round to question her, he found that she alone remained in the room with him. " Will you tell me whether you have noticed of late that your husband has been more despondent than usual? Or anything to lead you to suspect he was tired of life? And, had he access to any poisons?" he asked.

" I understand you," she answered. " You want to know if my husband was likely to have attempted his own life. I think you may at once dismiss the idea ; there is nothing more improbable ; he has been in quite his ordinary spirits, and always hopeful of ultimate recovery ; there was nothing within his reach, and he was unable to leave the room without assistance."

" You may be right, madam," he said, " but you should consider well what you say ; for, if it is not attempted suicide, it is attempted——"

" Stop ! " she cried, while her hitherto ashy face flushed crimson. " Don't speak the hateful word ! ! " and then, eagerly, she added, " Might there not have been something poisonous by accident in his food ! "

Mr. Tottenham shook his head, and answered " This is a case of poisoning by some narcotic."

Mrs. Spencer's manner betrayed great nervousness when she spoke again. " Perhaps I ought to tell you—though of course I know it could really have nothing to do with it—I *did* give my husband a dose of sleeping-draught, but he has had it often before, and it has never done him any harm, and it is with Dr. Marsh's sanction."

Again Mr. Tottenham scrutinized her delicate features, with their ever-changing colour ; and yet could form no conclusion, whether she was telling him a truth, or only half a truth.

Quick, heavy footsteps were heard ascending the stairs, and a diversion was occasioned by the entrance of Dr. Marsh, who, having returned from visiting a patient, had received a message that he was to follow to Mr. Spencer's house.

In another moment the two gentlemen were in deep consultation over the patient ; Mr. Tottenham giving a brief account of the case, and what treatment he had used ; and then a few murmured words followed, to which Dr. Marsh replied,

" Tut, tut, boy ! don't be so suspicious ! What mare's nest have you got hold of now? Why! she's the kindest, best, little woman in the place, and quite incapable of such an act."

" I shall never cease reproaching myself for having left him," wailed poor Mrs. Spencer. " I never dreamt any harm could happen to him, as I left him rather better than usual. You know Mrs. Lewis had a musical party at the Rectory this evening ; I had refused her invitation some days ago ; but this afternoon she wrote to say that the lady who was going to have played most of the accompaniments, was too ill to come ; and unless I would kindly fill her place, she did not know what she should do, as she had no one else who could read quickly at sight. I was *most* unwilling to go, but it seemed so ill-natured to refuse, and my faithful Mrs. Jennings, the children's nurse, said she would sit with Mr. Spencer till I returned. She is such an extremely nervous person, that I knew she would be terrified if one of his severe paroxysms came on ; so, to ensure him some quiet sleep, I gave him a good dose of the sleeping-draught before I called her up. But that could not have hurt him, could it, Dr. Marsh? "

" Certainly not, certainly not," replied the old gentleman. " You may make your mind quite easy on that score—that could not hurt him."

"No, *that* could not hurt him," re-echoed Mr. Tottenham, as he rose and left the room on some small pretext: for he was bent on finding the bottle containing the sleeping-draught, and on following up quickly any clue he could find to elucidate this mystery, as he considered it, before the scent became cold.

He passed into the dressing-room next door, where an array of medicine phials on a shelf had attracted his attention. On turning to close the door, he found himself followed by the faithful Mrs. Jennings, who, poor woman, appeared in a pitiful plight; her knees seemed shaking under her, and she clutched at the back of a chair for support, while ready tears were streaming down her cheeks as she spoke.

"Oh, sir, I couldn't tell you before the mistress; but yet I must say it, for I could not live with such a thing on my conscience—I am a wicked, *wicked* woman, and it is *I* that have killed the poor master. But, sir, if it was my last word, I declare I never meant it. It's for leaving him that I blame myself so, for if I had stayed as I was told, it would never have happened."

"Oh, you left him, did you?" said Mr. Tottenham, facing round upon her, "Tell me as briefly as you can the circumstances, and whether anyone had access to the sick-room in your absence?"

"I must tell my story in my own way, sir, or not at all," replied Mrs. Jennings, in an injured tone. "I have a sister who is going to emigrate, starting to-morrow for Liverpool; and we had agreed that to-night she should come and sup with me (which is not against the rules of the house). When the mistress called me up, and I saw how put out like she was at having to go to the party, I did not like to tell her about Jane coming; so I said I would stop with the master. But it seemed hard to have to give up saying good-bye to one's only sister, that I might never see again; so when the mistress was gone, I thought I might just slip downstairs for half-an-hour's chat, if I could get Araminta Mabel to stay in the room for that time. The young girl was not willing at first, but after a bit she consented; only I thought to myself that if the master was taken bad, she would not know what to do. Mrs. Spencer told me to give him a glass of whisky and water he always takes at bed-time; so I mixed it pretty strong, and, just to make sure that he would keep quiet, I put into it a good dose of the sleeping-draught he sometimes takes, and I told Araminta Mabel to give it to him a little later on—and why it should have gone and killed him to-night, when he has had it often before, is more than I can say," and her tears flowed afresh.

"And were you only half-an-hour away?" questioned Mr. Tottenham.

"It must have been rather more than that."

"An hour?"

"Well, sir, to be exact it must have been over an hour; you see when one gets chatting——"

"Yes, yes, I know. You say you gave 'a good dose.' Now was it *more* than a dose?"

"Yes, I suppose it *was* rather more."

"That will do, my good woman: go, and send Araminta Mabel Tomkins to me, I must question her;" and, as Mrs. Jennings withdrew, he rubbed his brow with a perplexed air, and said to himself: "Even so, it would hardly account for the condition I found him in."

In due time, Araminta Mabel presented herself; and she was in even more deplorable case than her predecessor. She threw herself on her knees, and rocked herself from side to side: and what with sobs, and cries, and ejaculations, it was some time before Mr. Tottenham could sufficiently pacify her to get anything like a coherent sentence from her.

"Do try to be calm, my good girl; do your best to collect yourself, and tell me whether you remained all the time in the room with Mr. Spencer, and whether anyone else came into it?"

"Oh dear! Oh dear!" she gasped; "I knew what you sent for me to say, and I may as well say it for myself, as hear it from you. Yes! you're quite right—I did it, miserable girl that I am—but I did not mean for to do it—nasty stuff that it was, to go and kill him—just to get me hung—when I didn't want to—and I daren't go home and tell father—he'll swing me hisself, will father, sooner than the disgrace of having the common hangman after me—"

"For goodness' sake, quiet yourself, and tell me at once what you mean. If you have been induced to take part in any dreadful crime for which you are sorry, the best thing is to confess at once what you have done, that we may have a chance of setting things right. Remember, Mr. Spencer is not dead, and perhaps we shall still be able to save him."

So, with many questionings and coaxings, and frequent symptoms of relapsing into her former inconsequent condition, Araminta Mabel unfolded her story:

"You see, sir, on Wednesday evenings—which this is Wednesday, sir—Peter, the gardener, from the big-house, passes by this way—Peter's my young man, please sir—and I was to be at the gate this evening when he went by, as he had something partickler to say to me. So when Mrs. Jennings called me to her, and told me as how she was obligated to go downstairs for a bit, and I was to sit and watch Mr. Spencer, and not leave the room till she came back, I was that put out, thinking of Peter, a-waiting and waiting down there, that I did not know what to do. And then I thought that no one need ever know, if I just stole away for ten minutes or so to have a word with Peter, and I could be back again before Mrs. Jennings was likely to go upstairs."

"And were you only the ten minutes?" inquired Mr. Tottenham.

"I don't want to tell no lies, sir. It must have been three times as much; for, when I got down, I found Peter had a *great deal* to say to me, and took a long time saying it too," said Araminta Mabel, an air of coquetry battling with her woe. "But oh, sir, it isn't *that*—it isn't so much that I left the po'r gentleman (though I didn't ought to), as what I did before I left."

More groans, more sobs, more coaxings, before Mr. Tottenham, whose patience was well-nigh exhausted, could elicit the fact.

"I was so afeard that the master would rouse hisself and p'raps call out, and so let on that I was gone; that to keep him quiet, I gave him the whiskey Mrs. Jennings had mixed, and I put into it some of the laudlum my sister gave me last week for the toothache—but it done *me* no harm, and only stopped the pain and made me sleep—so, why it should go and kill him, just for to get me hung!—Oh dear! Oh dear!"

No longer did the young man wait to listen to her lamentations. His curiosity had been satisfied much sooner than he anticipated. He had thought that by dint of patient inquiry he might have wrung a confession from *one* of the three women, and here, without an effort, it had been poured into his ear by all three; and two of them, with loud self-accusings, were ready to take up the *rôle* of murderess.

He was free once more to return to the sick-room, and, while assisting Dr. Marsh to the utmost, he narrated his discoveries to the astonished old gentleman.

It was nearly an hour later, when the two descended the stairs together, and sought Mrs. Spencer in her boudoir, where they were immediately followed by the other women concerned, with eager, questioning looks upon their faces.

Dr. Marsh cleared his throat in a deliberate manner, before he put an end to their suspense:

"I am happy to inform you all three, that our efforts to restore Mr. Spencer to consciousness have at length been crowned with success; and we have now left him almost as well as usual; though, between you, you had administered so large a dose of poison that even a stronger man might well have been put beyond the reach of our help by it. You have, I know, already suffered so much anxiety, that I think I need scarcely add any words of caution for the future, against this most dangerous amateur physicking. I should say it would be a long time before any of you dabble with narcotics again."

He then gave a few instructions to Mrs. Spencer, and the two doctors withdrew.

They had walked some distance down the street, when Dr. Marsh remarked:

"You are silent, Tottenham; what is it?"

"Of course, I am awfully glad we pulled the old fellow through," said the younger man, with a touch of regretfulness in his tone nevertheless. "But I can't help puzzling my mind over the legal bearings of the case, if he had slipped through our fingers. What could they have done, where individually each one was innocent in act, as well as intention, and yet collectively the three would have been guilty of manslaughter? And you see, now, we shall never know how it would have been."

THE END.

